# Complications and Functional Results of Surgery for Locally Advanced Prostate Cancer

**DOI:** 10.1155/2012/706309

**Published:** 2012-01-12

**Authors:** S. G. Joniau, A. A. Van Baelen, C. Y. Hsu, H. P. Van Poppel

**Affiliations:** Department of Urology, University Hospitals KULeuven, Herestraat 49, 3000 Leuven, Belgium

## Abstract

The role of surgery in clinical stage T3 prostate cancer (cT3 PCa) is still subject to debate. We reviewed the records of 139 consecutive patients who underwent a radical prostatectomy (RP) for cT3 PCa with a mean follow-up of 8 years. All data related to surgical and perioperative complications were collected. Continence and erectile function were assessed at 12 months postoperatively and long-term oncologic outcomes were analyzed. Rectal injury and injury of the obturator nerve occurred both in 0.7% of cases. No serious in-hospital complications were noted and no reintervention was needed. Lymphatic leakage was noted in 2.2% of patients and 1.4% experienced prolonged drainage of urine. In 7.2%, wound-related problems occurred. Anastomotic stricture occurred in 2.9%. These complication rates were not different compared to surgical series of RP in localized PCa. At 12 months, complete continence was 87.8% and erectile function had fully recovered in 6% and 10% of patients who underwent a non-nerve sparing or unilateral nerve-sparing procedure, respectively. 10-year estimated biochemical PFS, clinical PFS, CSS and OS were 51.8%, 85.6%, 94.6% and 85.9%, respectively. In cT3 PCa, RP is technically feasible with morbidity comparable to RP in clinically localized PCa. Long-term oncologic control was excellent.

## 1. Introduction

Locally advanced prostate cancer (PCa) is defined as cancer that has extended clinically beyond the prostatic capsule with invasion of the pericapsular tissue, the sphincter muscle, bladder neck, or seminal vesicles, but without lymph node involvement or distant metastases [1]. Locally advanced PCa is referred to as clinical stage T3-4 N0 M0 disease. T-staging is mainly based on the findings of digital rectal examination, while transrectal ultrasound, PSA level, PSA density, and the extent of cancer in prostate biopsies may provide additional information [2]. In a recent population-based Swedish study, 18.6% of prostate cancers presented as locally advanced, nonmetastatic PCa [3]. In another recent paper, based upon data from the SEER (Surveillance, Epidemiology, and End Results) database, between 11.6% and 15.3% of the patients presented with cT3 N0 M0 PCa, while 8% to 10.9% presented with T4 and/or N1 and/or M1 PCa [4]. These data from Europe and the US provide an estimation of the incidence of cT3-4 PCa, which is thought to be between 15 and 25%.

The optimal treatment of cT3 PCa has been subject to intense debate during recent years. According to the guidelines of the European Association of Urology (EAU), watchful waiting, radiation therapy (RT), Radical prostatectomy (RP), hormonal therapy (HT), and various combinations are valuable options to consider, depending on the general health status of the patient and the local extent of the tumour [5].

Many experts consider an RP for cT3 PCa a valid treatment option with excellent oncological outcome, but it is felt to be a burdensome procedure even for a skilled surgeon and feasibility has been questioned in the past.

In order to better define the place of surgery in cT3 PCa, we have conducted a retrospective study in 139 patients who underwent an RP for cT3 PCa. The patient files were critically reviewed and all data related to surgical and peri-operative complications were carefully collected. All data were compared to major contemporary series of RP in clinically localised disease. Additionally, functional results with respect to erectile function and continence were collected at 12 months postoperatively and long-term oncologic outcomes were assessed.

## 2. Material and Methods

From January 1997 to December 2003 we performed an RP with bilateral pelvic lymphadenectomy in 139 patients with cT3 PCa. Ultrasound guided prostate biopsies showed a median Gleason score of 7 (range 2–10). Prostate biopsy was performed in accordance with the random systematic octant biopsy technique: lateral systematic sextant biopsies with additional bilateral transition zone biopsies [6]. Additional biopsies were directed to the sites of abnormal digital rectal examination and abnormal transrectal ultrasound findings. Local staging was routinely performed by digital rectal examination and transrectal ultrasound. In 16 patients, endorectal coil magnetic resonance imaging was included to refine the local staging. Lymph node status was examined through a contrast-enhanced CT scan of the pelvis (*n* = 122) or an MRI scan (*n* = 4). Distant metastases were excluded by a bone scan (*n* = 123). In patients with PSA <10 ng/mL and a biopsy Gleason score <7, N and M staging was not performed, as the risk for nodal involvement in this group is estimated to be very low (≤4%) [7]. 125 patients (89.9%) were staged cT3a N0 M0 and 14 (10.1%) cT3b N0 M0 (Table 1).

As described earlier, our surgical technique focuses on clean apical dissection, neurovascular bundle resection at least at the tumour bearing site, complete resection of the seminal vesicles, and in some cases resection of the bladder neck [8]. In 129 patients (92.8%), a bilateral non-nerve-sparing RP was performed. In only 10 patients (7.2%), a unilateral nerve-sparing procedure was possible. In 10 patients (7.2%) a lymphadenectomy was not performed because of previous pelvic surgery or a low PSA level (<10 ng/mL) associated with a biopsy Gleason score <7.

In the peri-operative period, low molecular weight heparin and compression stockings were administered as thromboembolic prophylaxis. Postoperative pain was managed for 2 days by epidural patient-controlled anaesthesia. Oral ingestion and early mobilisation was encouraged from the first postoperative day. Patients were discharged after removal of all suction drains (as soon as drainage was fewer than 15 mL per 24 h), as soon as they were on a normal diet and were fully ambulatory and pain or discomfort was manageable by oral analgesia. The urethral catheter was left in situ at discharge and was removed during a one-night hospital stay at a mean of 12 days postoperatively. Since our group has shown that pelvic floor muscle exercises shorten the duration of incontinence and improve continence rates after an RP, physiotherapy was started at catheter removal [9]. Patients who remained incontinent at 1 year were offered the possibility of an artificial urethral sphincter implant.

At 6 to 8 weeks postoperatively, patients were reassessed for the first time and serum PSA was measured. For the first postoperative year, patients were seen at 3-month intervals. For the second and third years, patients were reevaluated every 4 months and 6 months thereafter.

Patients who underwent a unilateral nerve-sparing procedure were offered treatment with 5-phosphodiesterase-inhibitors, or intracavernous prostaglandin E2 injections if the obtained effect was insufficient. Patients who underwent a non-nerve-sparing operation were offered treatment with intracavernous injections.

Further treatment strategy was based upon final histopathology and PSA evolution. In case of positive surgical margins, patients were randomised according to the EORTC 22911 protocol to receive adjuvant pelvic irradiation or not [10]. In case of positive lymph nodes, early endocrine treatment was initiated. Invasion of the seminal vesicles with negative surgical margins was not an indication for early adjuvant therapy. A slowly rising PSA (PSA doubling time >12 months) in the absence of positive surgical margins or positive lymph nodes was interpreted as local relapse for which the patient was treated with pelvic irradiation (60 Gy). A PSA persistence in the presence of negative surgical margins and any steep rising PSA (PSA doubling time ≤12 months) after a period of undetectable nadir were both considered a sign of occult metastasis. Therefore, these patients were treated with endocrine treatment.

## 3. Results

Patient characteristics are described in Table 1. Mean age of the patients was 61.8 years (SD 7.0). Mean PSA was 13.7 ng/mL (range <0.02–97.0). Mean follow-up of the study was 98 months (range 7–162). Twenty patients (14.4%) had undergone previous pelvic surgery: inguinal hernia repair in 19 and surgery for pelvic fracture in one. Twelve patients (8.6%) had received neoadjuvant HT prior to surgery. No patient had undergone pelvic radiotherapy. In our population of 139 patients, mean operative time was 105 minutes (range 50–180) with a mean blood loss of 558 mL (range 100–2100). The urethral catheter was removed at day 12 (range 10–15). Mean admission time was 12 days (range 5–27).

### 3.1. Complications and Functional Results

Preoperatively, no ureteral or vascular injury occurred. Operative complications included one sectioning of the obturator nerve (0.7%) and one rectal laceration (0.7%). Treatment consisted of microsurgical repair of the obturator nerve and primary closure of the rectal laceration in a double layer. Long-term evolution was uneventful in both cases. No peri-operative mortality was noted.

In the peri-operative period no ureteral obstruction or urinary retention occurred. In 10 patients (7.2%) healing of the abdominal wound was delayed: 6 wound infections (4.3%) and 4 partial wound dehiscences (2.9%) occurred. Prolonged drainage in the suction drains was noted in 5 patients. Lymphatic leakage was present in 3 cases (2.2%). Two patients (1.4%) had a urinary leakage for 36 hours which resolved spontaneously with permanent suction. Prolonged drainage did not show to be prognostically relevant since all 5 patients obtained total continence at 12 months. All above mentioned patients were discharged without reintervention.

When lower urinary tract symptoms were present, a uroflowmetry was performed: within 12 months, 4 patients (2.9%) were diagnosed with an anastomotic stricture. One patient complained of a painful orgasm. Urethroscopy visualised a surgical clip at the level of the anastomosis. After removal of the clip, the dysorgasmia disappeared.

At 12 months, 98 patients were completely continent (70.5%) and 24 patients mentioned an occasional loss of a drip (17.3%). Incontinence for which protective pads were needed was only seen in 17 patients (12.2%). Of these 17 patients, one had already been treated for overactive bladder. Only 6 of these 17 patients needed more than one pad per day (4.3%). And only 2 of them complained of continuous and uncontrollable incontinence: an artificial urinary sphincter was therefore implanted (1.4%). Postoperative potency was evaluated at 12 months. 129 patients were treated by a non-nerve-sparing RP. 83.6% mentioned absence of erections; 10.4% experienced some tumescence, but not sufficient for vaginal intercourse, and 6% patients had erections, sufficient for successful vaginal intercourse. Mean age of these last patients was only 54.5 years (range 49.8 to 62.2 years). In the 10 patients who were treated with a unilateral nerve sparing procedure, erections did not recur in 40% and did recur partially though insufficiently for vaginal intercourse in 50%; 10% regained full erectile function.

Table 2 [11–16] compares the operative characteristics, peri-operative complications and mortality, late postoperative complications, and functional results of our present series of RP in locally advanced PCa with major series of RP in clinically localized PCa [11–15] and 1 series of RP in locally advanced PCa (Lerner) [16]. Mean blood loss ranged from 600 to 872 mL in the organ-confined series, which compares favourably with our series (558 mL) and the series by Lerner (945 mL). Rectal, ureteral, and obturator nerve injury occurred in 0.3–4.9%, 0.1–0.8%, and 0.3–1.6%, respectively, in the organ-confined PCa series. These results again compare favourably with the present series (0.7%, 0%, and 0.7%, resp.). In the series by Lerner, only rectal injury was mentioned (1.8%), while ureteral and obturator nerve injuries were not. Wound problems ranged from 0.9 to 13.8%, while reinterventions were rare at 0.5–1.7%. Again, this was not different in our series (7.2% and 0%, resp.) and the series by Lerner (2.7% and NA, resp.). Nonsurgical complications varied, but were infrequent, both in the literature reviewed as in the present analysis. Long-term complications (measured at 12 months) were mainly anastomotic strictures (range 0.7–13.8%) and incontinence, requiring pad use (12–20%). These were comparable to our series (2.9% and 12.2%, resp.) and the Lerner series (9.2% and 22.1%, resp.).

### 3.2. Oncologic Outcomes

At final histopathology, in 19 patients, positive surgical margins were found (13.7%). Of these specimens with positive surgical margins, 2 tumours were organ confined (pT2), 12 showed extraprostatic extension (pT3a), 4 were invading the seminal vesicles (pT3b), and one had invaded the bladder neck (pT4).  Table 3 provides an overview on the percentage of positive section margins according to the pathologic stage. In 14 patients, positive lymph nodes were found (10.1%). 13 were staged as clinical N0 by contrast-enhanced CT scan (*n* = 12) or MRI scan (*n* = 1). In one patient, preoperative lymph node staging was not performed because of PSA <10 ng/mL and biopsy Gleason score <7.

Postoperative evaluation included history, physical examination, and serum PSA measurement. PSA persistence (>0.02 ng/mL) at first follow-up was found in 14 patients (10.1%). These cases were considered surgical failures. In 10 of these 14 patients (71.4%), final histopathology revealed positive surgical margins or positive lymph nodes. Within one year, 10 patients (7.2%) underwent RT of the pelvis and 19 patients (13.7%) were started on endocrine treatment because of positive surgical margins, PSA persistence, or rising PSA (Table 1). At a mean follow-up of 98 months (median 98, range 7–162), 35.5% of the patients had received adjuvant or salvage RT and 38.8% of the patients had received adjuvant or salvage HT.

**Figure 1:**
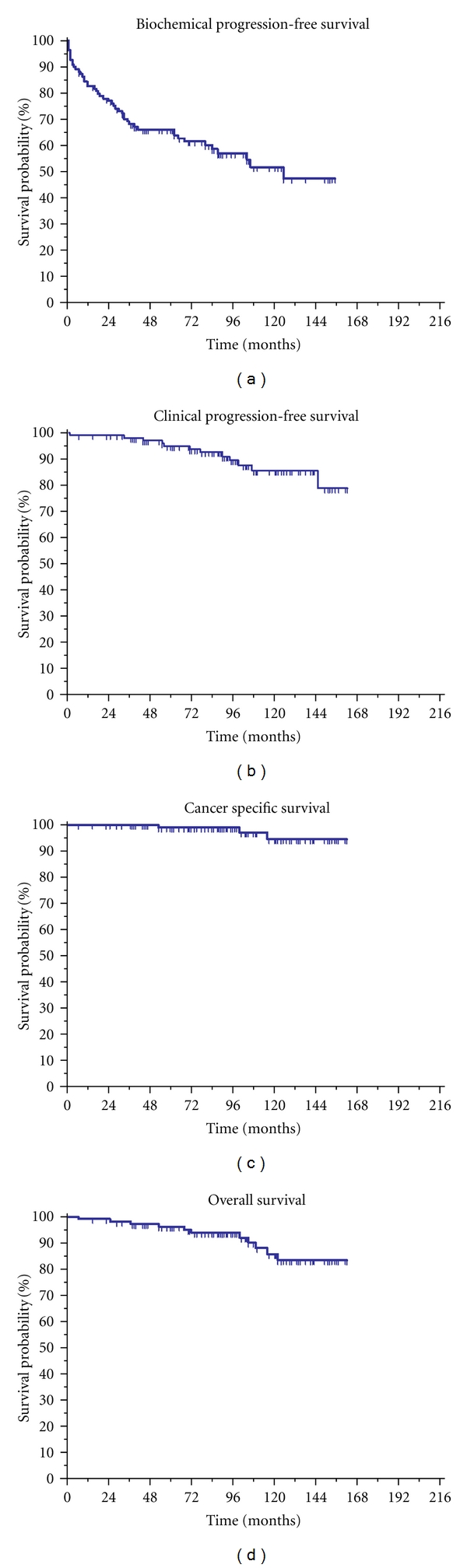
Kaplan-Meier plots for the oncologic outcomes of surgery for cT3a-b PCa. (a) Biochemical progression-free survival. (b) Clinical progression-free survival. (c) Cancer-specific survival. (d) Overall survival.

The long-term oncologic outcomes were assessed by Kaplan-Meier survival estimates. The 10-year estimated biochemical progression-free survival, clinical progression-free survival, cancer specific survival, and overall survival rates were 51.8%, 85.6%, 94.6%, and 85.9%, respectively, (Figures 1(a)–1(d)).

## 4. Discussion

Treatment options for locally advanced PCa vary and the jury is still out regarding the optimal treatment [17]. Watchful waiting, RT, HT, surgery, and combinations have been proposed.

In cT3 PCa, Thompson reported a 60 to 70% 5-year overall survival with watchful waiting [18]. Similarly, Johansson et al. mention a 15-year progression-free survival rate of 46.6% and a disease-specific survival rate of 56.5% [19]. cT3 PCa is therefore regarded as a significant tumour with a considerable associated mortality, especially in patients with a long life expectancy. Thus, watchful waiting is only allowed in a strict minority of selected patients with a poor general health status [18, 19].

Until the early eighties, radiotherapy was the treatment of choice for localized and locally advanced PCa. With radiotherapy as monotherapy, 10-year disease-free survival rates of 19–44% and overall survival rates of 21–54% have been reported [20–23]. At 25-year follow-up, radiotherapy as monotherapy only added a neglectable gain in survival. When patients did not die of intercurrent disease, they were highly likely to develop recurrence and to die of PCa [20].

In an attempt to improve disease-free survival and overall survival, the combination of RT and HT was evaluated. Laverdiere et al. had indeed shown a significant improvement in oncological outcome with adjuvant HT [24]. These findings were corroborated in randomised trials of the European Organisation for Research on Treatment of Cancer (EORTC) and the Radiation Therapy Oncology Group (RTOG) [25, 26]. The EORTC trial 22863 turned out to be a milestone study. Disease-specific survival and overall survival rates at 5 years improved from 79% to 94% and from 62% to 78%, respectively, in favour of combined RT and HT. Neoadjuvant HT was evaluated in the RTOG 86-10 trial. A significant decrease in local and distant progression and a significant increase in disease-free survival and disease-specific survival were noted at a mean follow-up of 8 years. However, overall survival did not increase significantly [27].

By many, the combination of external-beam RT and adjuvant HT is since considered a standard therapeutic option in patients with cT3 PCa.

Literature on the value of RP as an option for cure in cT3 PCa is limited. However, clinical evidence showing 5-year disease-specific survival rates ranging between 85% and 100% is available [28–31]. Additionally, RP can prevent local tumour-associated complications and provide a clear definition of failure after therapy compared to the more vaguely defined failure parameters after RT. Furthermore, overstaging of cT3 PCa ranges from 13 to 27% (pT2) (Table 4) [16, 28, 29, 32–34]. In pT2, RP has a very high chance of cure and long-term outcome after RP is very good [34].

Some locally advanced PCa will not be cured by surgery alone, and therefore, combinations with hormone therapy or radiotherapy have been investigated. Neoadjuvant HT did not improve biochemical or clinical progression, nor survival rates in RP [35–37]. Adjuvant HT after RP has shown to be beneficial, especially in poor prognosis disease [16]. Early adjuvant RT has also shown a lower risk of local recurrence, a longer time to progression, and an improved cancer-specific and overall survival [10, 38]. This effect was also more pronounced in high-risk patients: EORTC trial 22911 showed a clear improvement of progression-free survival and local control in patients with positive surgical margins or pT3 prostate cancer when RP was combined with RT [10]. Recently, long-term follow-up of cT3 PCa treated primarily with a prostatectomy has been published. Majority of patients underwent adjuvant RT and/or HT. 5-, 10-, and 15-year disease-free survival and disease-specific survival rates were 85%, 73%, and 67% and 95%, 90%, and 79%, respectively [33].

The general impression is that complications such as rectal injury, haemorrhage, deep venous thrombosis, pulmonary embolism, urinary fistula, ureteral obstruction, stress incontinence, impotence, anastomotic stricture, and peri-operative death are more common in the cT3 patient group. Our review of literature shows that the mortality risk associated with RP is merely a theoretical risk. Other surgery-related complications such as rectal injury, ureteral obstruction, and injury to the iliac vessels or obturator nerves are encountered rarely and do not account for a significant amount of morbidity. At an incidence between 0.6% and 7.3%, all of these per-operative complications could be resolved during the same operation. Long-term consequences such as anastomotic strictures occur in 0.7% to 13.8% of patients. One single dilatation has a success rate of up to 75% [12, 14]. Another late problem is incontinence. In 12% to 22.1% of patients, at least one protective pad is still needed at 12 months [11–16, 33].

In Table 2, we compare the complication rates and functional results of our series of 139 cT3 PCa patients with some major contemporary RP series in organ-confined and 1 series of RP in locally advanced PCa. Postoperative complications are grouped according to organ system. With absent mortality, a peri-operative complication rate of 1.4%, and postoperative complication rate of 12.9%, our cT3 population is exposed to an equal risk of complications compared to patients who undergo an RP for cT1 or cT2 tumours. At the same time, our results compare favourably with those mentioned by Lerner in RP for locally advanced PCa. The only paper which has so far directly compared surgical complications in locally advanced PCa versus localized disease in a single institution is from Gontero et al. The two groups did not differ significantly in surgical morbidity except for blood transfusion, operative time, and lymphoceles, which showed a higher rate in patients with advanced disease [39]. We corroborate these results in our present analysis.

Furthermore, in our series, functional results at 12 months show total continence (no pad necessary) in 87.8% and socially acceptable continence (max. 1 precautionary pad) in 94.2%, which is well within acceptable ranges. Finally, anastomotic stricture was encountered at a rather low rate of 2.9%. Expectedly, potency rates were poor in non-nerve-sparing RP (6% full recovery at 1 year and 10% partial recovery), while those rates were better in unilateral nerve-sparing RP (10% full recovery and 50% partial recovery). As complete recovery of erectile function can take up to 36 months, further improvement of these results may be expected [40]. Furthermore, modern imaging (Magnetic Resonance Imaging (MRI), diffusion-weighted MRI) allows more accurate preoperative assessment of tumour invasion in the neurovascular bundle, further increasing the indications for a nerve-sparing approach [41].

Surgical margins after RP are of great importance in progression and oncological outcome [42–46]. Margin positive status varies between 29% and 60.5% in the corresponding articles. The lowest incidence of positive margins was 29% and was found in a population of predominantly organ-confined PCa (73.7% pT2) [42]. In our series of cT3 PCa, 31.1% were pT2. Positive surgical margins were found in only 13.7%, which is the lowest rate in literature to our knowledge. It is clear, though, that surgery for locally advanced PCa had a considerable learning curve. At our institution, the learning curve translated into a dramatic decrease in positive margin rates from 66.7% in the period 1987–1994 to 43.3% in the period 1995–1999 to 10.0% in the period 2000–2004 [7].

Our present analysis is not devoid of limitations. First, this is a retrospective analysis of complications and functional results, using data extracted from patient files. Inherent biases are to be expected, as sometimes more discrete complications can be missed. Second, preoperative data on the functional status of the patient were not collected, limiting the interpretation of the results. Third, the complications and functional results were compared to data extracted from the literature. Indeed, a more solid approach would be to prospectively compare data on RP in cT3a-b PCa with data on RP in localized disease from the same institution. Nevertheless, we believe that our analysis has its value in outlining the incidence of complications and the functional results that can be expected after RP for locally advanced PCa. It has to be stressed that data on this subject are extremely scarce. Finally, a significant number of patients received adjuvant or salvage RT and/or HT treatment following surgery, limiting the interpretation of the results regarding the value of surgery in locally advanced PCa. Accepting this limitation, oncologic control with RP as a first step in the treatment of locally advanced PCa is excellent.

## 5. Conclusion

Our experience with 139 patients confirms the surgical feasibility of RP for cT3 PCa, showing complication rates comparable with RP in organ-confined PCa and showing a very low incidence of positive surgical margins and associated failure of surgery. Improvement can be expected by further defining the patient population most suitable for surgery and by further optimising adjuvant treatments such as RT and HT. Continence rates were also comparable with those achieved after RP for localized PCa. A nerve-sparing approach was only considered possible in a limited number of patients. It has to be expected, though, that modern imaging will further increase the indications for nerve-sparing surgery in locally advanced PCa.

Prospective randomized clinical trials are needed to compare oncological outcome, treatment-related complications, and quality of life in the different treatment options for cT3 PCa.

## Figures and Tables

**Table 1:** Patient characteristics.

Number of patients	139

Age (years), mean (±SD)	61,8 (±7,0)

cT3a	89,9% (*n* = 125)
cT3b	10,1% (*n* = 14)
Biopsy Gleason score, median (range)	7 (2–10)
PSA (ng/mL), mean (range)	13,73 (3,1–97,0)

Previous surgery	14,4% (*n* = 20)
Neo-Adjuvant Androgen Deprivation Therapy	8,6% (*n* = 12)
Non-nerve-sparing procedure	92,8% (*n* = 129)

Unilateral nerve sparing procedure	7,2% (*n* = 10)
Lymphadenectomy not performed	7,2% (*n* = 10)
Hospital stay (days), median (range)	12 (5–27)

pT2	31,1% (*n* = 42)
pT3a	51,1% (*n* = 69)
pT3b	16,3% (*n* = 22)
pT4	1,5% (*n* = 2)

PSA persistence	10,1% (*n* = 14)
Pathological Gleason score, median (range)	7 (4–9)
Pathological node positive	10,1% (*n* = 14)
Surgical margin positive	13,7% (*n* = 19)

Adj radiation therapy within 1 year	7,2% (*n* = 10)
Adj endocrine therapy within 1 year	13,7% (*n* = 19)

**Table 2:** Complication rates after open radical retropubic prostatectomy.

	Joniau	Dillioglugil et al. [11]	Hisasue et al. [12]	Gaylis et al. [13]	Maffezzini et al. [14]	Lepor et al. [15]	Lerner et al. [16]
Number of patients	139	472	123	116	300	1000	812

cT1 % (pT1 %)	0	20.3	44.7	43	(0)	78.5	0
cT2 % (pT2 %)	0	72.7	55.3	57	(66.4)	21.3	0
cT3 % (pT3 %)	100	6.9	0	0	(29.9)	0.2	100
Mean age (years)	62.0	63	66	66.6	65.5	60.3	

Mean operation time (min)	105			155			
Mean blood loss (mL)	558			872	600		945

Mortality %	0			0	0		0.4
Rectal injury %	0.7	0.6	4.9	0.9	0.3	0.5	1.8
Ureteral injury %	0	0.2	0.8		0.3	0.1	
Iliac vessel injury %	0	1.1					
Obturator nerve injury %	0.7	0.2	1.6		0.3		

Angor/myocardial infarction %	0.7	1.7				0.6	0.4
Other cardiac complications %	0	10.6	0.8			0.2	
Pulmonary complications %	0	3.8				0.1	
Deep venous thrombosis/pulmonary embolism %	0	2.3	0.8	3.4	0.3	0.3	4
Gastrointestinal complications %	0	5.1	0.8			0.6	
Neurological complications %	1.4	1.5				0.2	
Other infectious complications %	0	4.7	0.8				0
Prolonged drainage (urine, lymph, blood) %	3.6	2.8	8.9		2	0.7	0.8
Acute retention %	0	0.6			2		
Reintervention %	0				1.7	0.5	
Woundproblem %	7.2	3.0	13.8	0.9	1	0.8	2.7

Anastomotic stricture at 12 months %	2.9		13.8		0.7	1	9.2
Not dry (in need of pads) at 12 months %	12.2		12.7	20	12		22.1

**Table 3:** Comparison between positive surgical margins and pathologic staging after radical retropubic prostatectomy.

	Positive surgical margins
All patients	13.7%
pT2	4.8%
pT3a	17.4%
pT3b	18.2%
pT4	50%

**Table 4:** The percentage of overstaging and understaging in clinical locally advanced T3 prostate cancer.

Authors	pT2	pT4/N+
Van Poppel et al. [28]	13%	8%/11%
Van den Ouden et al. [29]	15%	3.4%/15.6%
Lerner et al. [16]	17%	—/33%
Morgan et al. [32]	22%	42% (stage D1)
Ward et al. [33]	27%	—/27%
